# Functional connectivity and quality of life in young adults with cerebral palsy: a feasibility study

**DOI:** 10.1186/s12883-020-01950-7

**Published:** 2020-10-23

**Authors:** Diana Tajik-Parvinchi, Andrew Davis, Sophia Roth, Peter Rosenbaum, Sarah N. Hopmans, Aya Dudin, Geoffrey Hall, Jan Willem Gorter, MyStory Study Group, Jan Willem Gorter, Geoff Hall, Peter Rosenbaum, Darcy Fehlings, Mark Ferro, Andrea Gonzalez, Sidney Segalowitz, Christine Lackner, Robert Palisano, Diana Tajik-Parvinchi, Sarah Hopmans, Dayle McCauley, Aya Dudin, Sophia Roth, Andrew Davis

**Affiliations:** 1 Department of Pediatrics and CanChild, McMaster University, Hamilton, Ontario L8S 1C7 Canada; 2 Department of Psychology, Neuroscience & Behaviour, McMaster University, Hamilton, Ontario L8S 1C7 Canada; 3 School of Rehabilitation Science, Faculty of Health Sciences, McMaster University, 1280 Main Street West, Hamilton, Ontario L8S 4K1 Canada

**Keywords:** Functional connectivity, Cerebral palsy, Adults, fMRI, Resting state

## Abstract

**Background:**

Cerebral Palsy (CP) is a group of disorders that affect the development of movement and posture. CP results from injuries to the immature brain during the prenatal, perinatal, or postnatal stage of development. Neuroimaging research in CP has focused on the structural changes of the brain during early development, but little is known about brain’s structural and functional changes during late adolescence and early adulthood, a period in time when individuals experience major changes as they transition into adulthood. The work reported here served as a feasibility study within a larger program of research (*MyStory* Study). We aimed to determine whether it would be feasible to scan and obtain good quality data without the use of sedation during a resting state condition for functional connectivity (FC) analyses in young adults with CP. Second, we aimed to identify the FC pattern(s) that are associated with depressive mood ratings, indices of pain and fatigue, and quality of life in this group.

**Methods:**

Resting state functional images were collected from 9 young people with CP (18–29 years). We applied a stringent head motion correction and quality control methods following preprocessing.

**Results:**

We were able to scan and obtain good quality data without the use of sedation from this group of young individuals with CP who demonstrated a range of gross motor ability. The functional connectivity networks of interest were identified in the data using standard seed regions. Our analyses further revealed that higher well-being scores were associated with higher levels of FC between the Medial Pre-Frontal Cortex and the right Lateral Parietal regions, which are implicated in prosocial and emotion regulations skills. The implications of this association are discussed.

**Conclusion:**

The findings of the present study demonstrate that it is feasible to conduct resting state functional connectivity in young adults with CP with different gross motor abilities without the use of sedation. Our results also highlight a neural circuitry that is associated with the self-report of quality of life and emotion regulation. These findings identify these regions/circuitries as important seeds for further investigations into mental health and wellbeing in CP.

## Background

Cerebral Palsy (CP) is characterized by a group of disorders that affect the development of movement and posture. CP appears to result from injuries to the immature brain during the prenatal, perinatal, or postnatal stage of development [1]. In addition to motor movement difficulties, individuals with CP may also experience impairments of sensation, perception, cognition, communication, and behaviour through epilepsy and/or other secondary musculoskeletal difficulties [1]. Brain injury in CP can involve widespread reduction of white and grey matter volumes as well as altered connectivity in various neural networks [2]. Many earlier neuroimaging studies in CP have focused on the structural changes of the brain during early development, but much less is known about brain’s structural and functional changes in adolescents and adults with CP.

During the transition time between adolescence and adulthood, all adolescents – with or without CP – undergo developmental challenges associated with growing up. Adolescents and young adults with CP experience these challenges as well as those related to their physical and mental health. Prior research suggests that during this time individuals with CP engage in more passive, solitary activities, which can affect self-concept and sense of belonging [3–5]. They are less likely to pursue post-secondary education, have low employment rates, and take part in fewer social activities [6–8]. In order to ease the transition from adolescence to adulthood for young people with CP, it is important to gain a better understanding of the relationship between the maturing brain and the physical, psychosocial, and cognitive factors that influence quality of life and wellbeing.

Functional connectivity (FC) is defined as the temporal correlation in the Blood Oxygen Level Dependent (BOLD) signal between separable disparate localized brain regions, as measured with functional magnetic resonance imaging ([9]; fMRI). Biswal et al. [10] noticed that the difference between the task-induced BOLD signal and the signal change from a baseline condition was only 1-5%. This finding revealed the substantial amount of activity in the human brain at rest and sparked the interest of researchers in “resting state” studies [11]. Resting state fMRI provides a method of exploring this baseline activity of the brain. During resting state conditions, participants are not required to carry out any tasks; they are simply asked to fixate on a central fixation point on a screen or to close their eyes and rest. As a result, resting state acquisitions are less demanding than task-based paradigms and can be of particular use when studying children or clinical groups [12, 13]. This method also has the added benefit of avoiding the controversies related to the role of cognitive effort in performance [13]. However, recent findings indicate that resting state FC is very sensitive to motion artifacts and even small transient head motions can introduce false correlations in resting state FC analysis [14] making head motion a major source of error. This becomes a concern when considering resting state FC in individuals with CP given the associated physical and motor challenges in CP and the fMRI demand to remain still in the scanner bore. Although resting state FC holds much promise in neuroimaging research, it is not clear whether this imaging method would be feasible in individuals with CP and provide meaningful data.

The human brain is organized into a number of interacting functional networks [15, 16]. During typical development functional connectivity increases between specific brain regions that make up these brain networks [17]. Over time, these networks show increased within-network connectivity and higher levels of functional segregation from one another [17]. Many networks have been identified and shown to behave in a complementary manner to one another, with one “switching on” while the other “switches off”. The networks examined in the present study are: the Dorsal Attention (DA) network, the Default Mode Network (DMN), the Salience Network (SN), the Frontal-Parietal network, the SensoriMotor network, the Language network, and two Cerebellar regions (Appendix A). The DA and the DMN are functionally competitive networks with the former supporting externally directed cognition and the latter guiding the internally driven cognition [18, 19]. The DMN is commonly active during passive or low demand tasks such as rest. The frontoparietal network has been suggested to serve as a mediator between the DA and DMN and function as a support to goal-directed behavior [20, 21]. The SN (particularly the Anterior Insula) appears to be an important network involved in orchestrating the switch between brain networks and is responsible for detection of saliency and direction of attention [22].

Individuals with CP often experience chronic pain [23]. Chronic pain can result in pain-related anxiety and reduced quality of life [24]. The experience of pain appears to disrupt activities associated with the DMN [25, 26], with brain structures commonly associated with the salience network such as the ACC and the anterior insula [24, 27], and with cognitive performance [28]. We currently do not know whether living with chronic pain is associated with altered connectivity pattern(s) in individuals with CP. Also, given the higher incident of depression in individuals with CP [29], it is currently unclear whether depressive symptoms are associated with altered connectivity patterns in CP. Increased functional connectivity between subgenual prefrontal cortex and the DMN has been shown in individuals with major depression [30, 31]. To date, no studies have examined functional connectivity in young adults with CP, and we do not know the association between functional connectivity and mental health factors that may affect quality of life such as depressive symptoms, chronic pain, and fatigue in these young individuals.

There have been relatively few neuroimaging studies in adults with CP and to our knowledge no studies that have examined FC in a resting state condition in this population. The only study that has investigated resting state FC was conducted in children with CP who were sedated in order to control for motion artifacts [32]. Qin et al. [32] reported altered FC in several neural networks including the sensorimotor, left frontoparietal, and the SN networks that may be related to motor and cognitive difficulties in children with CP. They also reported distinct FC differences between the spastic and dyskinetic subtypes of CP. Other studies have focused on structural differences, implementing task-based paradigms [33–36]. Fiori et al. [34] applied tactile stimulation to the thumb and the index finger of each participant and examined somatosensory activation in adolescents and young adults with CP. However, they did not perform group level analyses on their 6 participants with CP and only carried out group level analyses in their participants without CP. Their single participant examination demonstrated individual differences in reorganization of somatosensory cortex in response to the tactile stimulation that was associated with hemiparetic side and severity of the tactile impairment. Hilderley et al. [35] only had 4 children with CP and examined motor cortical activation during ankle movement to contribute to our understanding of cortical activation for intervention purposes. Van de Winckel et al. [36] examined somatosensory brain activation during passive somatosensory discrimination tasks in 16 adolescents and young adults with CP to investigate the benefits of rehabilitation efforts on the brain. They reported differences in brain activation as a result of somatosensory exercises between typically developing children and those with CP. Although research efforts are beginning to be directed to our understanding of structural alterations in adults with CP, there is still a dearth of studies available in resting state FC in adults with CP. Currently, it is not even clear whether this type of study would be feasible in participants with CP, since the only study that has implemented resting state FC sedated the children with CP [32].

The work reported here served as a feasibility study within a larger program of research entitled “Brain-Behaviour Correlates of Health and Well-being in Adolescents and Young Adults with CP” (short title: *MyStory* Study) as part of the Cerebral Palsy Integrated Neuroscience Discovery Network (CP-NET), an Integrated Discovery Program conducted in partnership with the Ontario Brain Institute in Ontario, Canada. We aimed to answer the following questions: 1) Will it be feasible to scan and obtain good quality resting state functional connectivity data in young adults with CP without sedation, given their motor and physical challenges and the associated expectations of fMRI scanning protocol such as lying down straight and remaining still in a scanner for some time; 2) Will we be able to identify the 6 networks of interest (DA, DMN, SN, the Frontal-Parietal network, the SensoriMotor network, and the Language network); 3) Are there connectivity pattern(s) that are associated with depressive mood ratings, indices of pain and fatigue, and quality of life in this population. Given the association between FC involving the DMN and major depression [30], we hypothesized that a positive correlation would be observed between depressive scores and the DMN (see Appendix A). Also, given prior associations between pain and certain regions in the SN network (ACC and anterior insula) [24, 27], we hypothesized that a correlation would be observed between pain severity and/or impact of pain on daily activities and the brain regions associated with SN (see Appendix A). However, insofar as this was a feasibility with additional exploratory analyses, no specific a priori hypotheses were formulated in terms of quality of life and fatigue.

## Methods

### Participants

Participants with a diagnosis of CP, age 16–30 years of age and of all gross motor ability levels (GMFCS level I-V), resident of Ontario, Canada, able to complete online surveys (with or without assistance), who took part in the larger study “*MyStory*” indicated their interest in the fMRI study by checking off a box on the consent form. Those who expressed interest were contacted regarding the fMRI scans. Additional inclusion criteria for the fMRI study consisted of being able to follow simple instructions, and lie flat with support for approximately 45 min. The exclusion criteria consisted of general contraindications to MRI (e.g., non-removable metal implants), or seizures not controlled by medication over the past 2 years. For this feasibility study, a total of 15 participants were screened of whom 5 were excluded due to contraindications to MRI and one participant did not complete the resting-state condition. The final sample included 9 participants with CP (18–29 years of age) with a range of GMFCS levels (Table 1). The Participants were recruited in accordance with the local research ethics board and all provided written consent.

**Table 1:** Demographic information

Participant	Gender	Prior Imaging Scans	GMFCS level	Sub-type	Generalized white matter loss	Focal white matter loss/encephalomalcia	Caudate nucleus	Lentiform nucleus	Brainstem	Other
1	F	No	1	Unilateral (left) Spastic	Mild right frontal and parietal centrum semiovale				Wallerian degeneration right midbrain and pons	
2	F	Yes	2	Unilateral (left) Spastic		Right frontal and parietal corona radiata; right posterior limb internal capsule			Wallerian degeneration right midbrain and pons	
3	F	No	1	Unilateral (right) Spastic	Bilateral symmetric parietal					
4	F	Yes	4	Bilateral Spastic	Bilateral assymetric parieto-occipital (L > R)	Left corona radiata				
5	M	No	1	Unilateral (left) Spastic		Right frontal and parietal corona radiata; left frontal and parietal corona radiata				
6	M	Yes	1	Unilateral (left) Spastic		Right posterior limb internal capsule and centrum semiovale	Right body	Right putamen		
7	F	Yes	2	Bilateral spastic						
8	M	Yes	4	Bilateral spastic	Bilateral symmetric severe parieto-occipital	Right frontal porencephalic cyst				VP shunt right
9	F	No	3	Bilateral spastic	Bilateral symmetric parieto-occipital					

*GMFCS* Gross motor function classification system

### Procedure

Participants filled out the questionnaires online through the *MyStory* REDCap website. REDCap is a secure web application for building and managing online surveys and databases *(**www.project-redcap.org**).* Once the participant provided written consent online, the study research assistant emailed them their unique, anonymize username and password, which they used to log in to the system. Once logged in, participants completed the surveys, with or without assistance from the user guide. If participants had questions, or difficulties completing the surveys, they were able to contact the research assistant by phone or email for help. We did not implement any strategies to increase scanning success rate since our study consisted of young adults and prior findings have made recommendations for younger children with the need to implement pre-scan preparations decreasing for older children and adolescents [37]. However some general guidelines and recommendations to familiarise children with the fMRI environment are available outside the CP field for those interested in scanning children with CP without the use of sedation [38–40].

### Measurements

We measured severity of CP in terms of motor function using the GMFCS [41]. The GMFCS includes five levels that are intended to represent meaningful differences in gross motor function, in mobility, varying from level I (most functional level) to level V (least functional level). Other self-reported measures for the present study addressed: fatigue ([42]; Fatigue Impact and Severity Self-Assessment; FISSA), depression ([43]; Center for Epidemiological Studies Depression Scale; CES-D), quality of life ([44]; Quality of Life Instrument for People with Developmental Disabilities; QoL), and the Pain Questionnaire pain [45]. FISSA is a 38-item scale to which participants respond to questions about fatigue using a 1–5 Likert scale (higher scores indicate greater fatigue) and open-ended questions. For the present study, the total scores for impact, severity, and management of experienced fatigue were used. CES-D is a 20-item self-report instrument that evaluates depressive symptoms defined by the American Psychiatric Association’s Diagnostic and Statistical Manual (DSM-IV) for a major depressive episode. Participants respond on a 4-point Likert scale, where higher scores indicate higher levels of depression. QoL is a 27-item questionnaire designed to assess the quality of life of people with developmental disabilities. Three instruments focus on the perspectives of the individual, a person who knows the individual well, and a trained assessor. We only collected information with regard to perspectives of the individual. The subscale of *Being* consists of physical, psychological, and spiritual well-being. Scores from these subscales were averaged to represent *Well-Being* and used in the present study to capture quality of life. Pain was assessed with a 3-item self-report instrument [45], which evaluates the frequency and severity of physical pain in individuals with CP.

### Image acquisition and procedure

All participants were screened for standard MRI contraindications. Scanning was performed at the Imaging Research Centre of St. Joseph’s Healthcare, Hamilton. The scanning protocol included task-based and resting state conditions. The entire scanning duration was about 45 min. In the current study, we report the results for the resting-state condition.

All images were acquired on a 3 T GE Discovery 750 system (GE Healthcare, Waukesha, WI, USA), running on software version 25 and using a 16 channel Head-Neck & Spine array receiver coil (GE Healthcare, Waukesha, WI, USA). Following localization, a 3D T1-weighted structural image (magnetization-prepared gradient echo) with 1 mm^3^ isotropic resolution was acquired (Sagittal orientation, FOV = 240 × 240 mm^2^; TR = 6.4 ms, TE = 2.8 ms, TI = 450 ms, flip angle (FA) = 15°, receiver bandwidth per pixel (BWpp) = 244.141 Hz, ARC acceleration with *R* = 2). Resting state functional images were collected using a T2*-weighted single-shot interleaved echo-planar gradient echo imaging sequence (Axial orientation, FOV = 256 × 256 mm^2^ 4 × 4 × 4 mm^3^ isotropic resolution, 36 slices with no slice gap; TR = 2000 ms, TE = 30 ms, FA = 75^o^, BWpp = 7812.5 Hz, ASSET acceleration with *R* = 2). Two hundred and forty volumes (total time 8 min) were collected while participants fixated on a cross (+) in the center of the display screen inside the scanner.

### fMRI preprocessing

Imaging data were preprocessed using the Conn toolbox ([46]; https://www.nitrc.org/projects/conn), which utilized SPM ([47]; v12; http://www.fil.ion.ucl.ac.uk/spm) running under Matlab (R2012a). The structural images were bias corrected, normalized to MNI space (ICBM 152 non-linear 6th Generation atlas), and segmented by tissue type using SPM’s algorithm to generate gray matter (GM), white matter (WM), and cerebralspinal fluid (CSF) masks for each participant. The functional images were first slice-timing corrected to account for differences in sampling times of interleaved fMRI slices. The fMRI time series were motion corrected using registration to mean volume in a two-stage process in order to quantify and correct for inter-scan movement. The functional images were co-registered with the T1-weighted structural scans and then normalized. Finally, the fMRI data were smoothed with an isotropic 5 mm FWHM Gaussian kernel.

#### Head motion

Recent studies have shown that frame-to-frame displacements influence data quality [14] and that even small transient head motions can introduce spurious correlations in resting state functional connectivity analyses [48]. In order to address this risk, in addition to motion correction in the pre-processing pipeline, we additionally applied a *scrubbing* method on the fMRI data to eliminate motion artifacts using conservative settings (0.5 mm frame-to-frame displacement, z-stat = 3 for frame-to-frame signal change) [14]. The ArtRepair toolbox was used to identify problematic frames for scrubbing (http://cibsr.stanford.edu/tools/human-brain-project/artrepair-software.html). The frames were effectively removed from the analysis using covariates (one per frame) in the 1st‑level analysis.

### Data quality assessment

We defined good quality data by examining the following criteria:

- Visual examination of the distribution of connectivity values (r) prior and after denoising to examine skewedness and shape of the distribution;
- The ability of the data to withstand volume censoring. The neuroimaging field accepts around 5 min of data as an acceptable quantity for restating state functional connectivity analyses [49].

### Data analysis

Functional connectivity analysis was carried out with the Conn toolbox. The denoising process included (i) regression of confounding signals from WM, CSF, motion parameters (6 DOF), and scrubbing; (ii) application of a 0.01‑ 0.08 Hz bandpass filter; (iii) linear detrending [10, 50]. We ran a Region of Interest to Region of Interest (ROI-to-ROI) connectivity analysis using bivariate correlation with HRF weighting among 28 functional and anatomical regions (see Appendix A for ROI details) using the Harvard-Oxford atlas as distributed with Conn [51]. The FC networks of interest were investigated using standard seed regions defined by previous literature and examining the correlation between the seed and the 28 ROIs.

At the group level analysis, scores from the self-reported measures with FISSA, CES-D, QOL, and Pain were entered as regressors in Conn. Four regression analyses were performed for each of these measures to examine the association between functional connectivity and the clinical measure. All 28 seeds were entered for each regression and if the regression was significant, pairwise comparisons were performed to identify the correlations responsible for the group effect. All *p*-values reported have been corrected for multiple comparisons using a false discovery rate (FDR) approach and thresholded at FDR-α = 0.05.

## Results

**Fig. 1:**
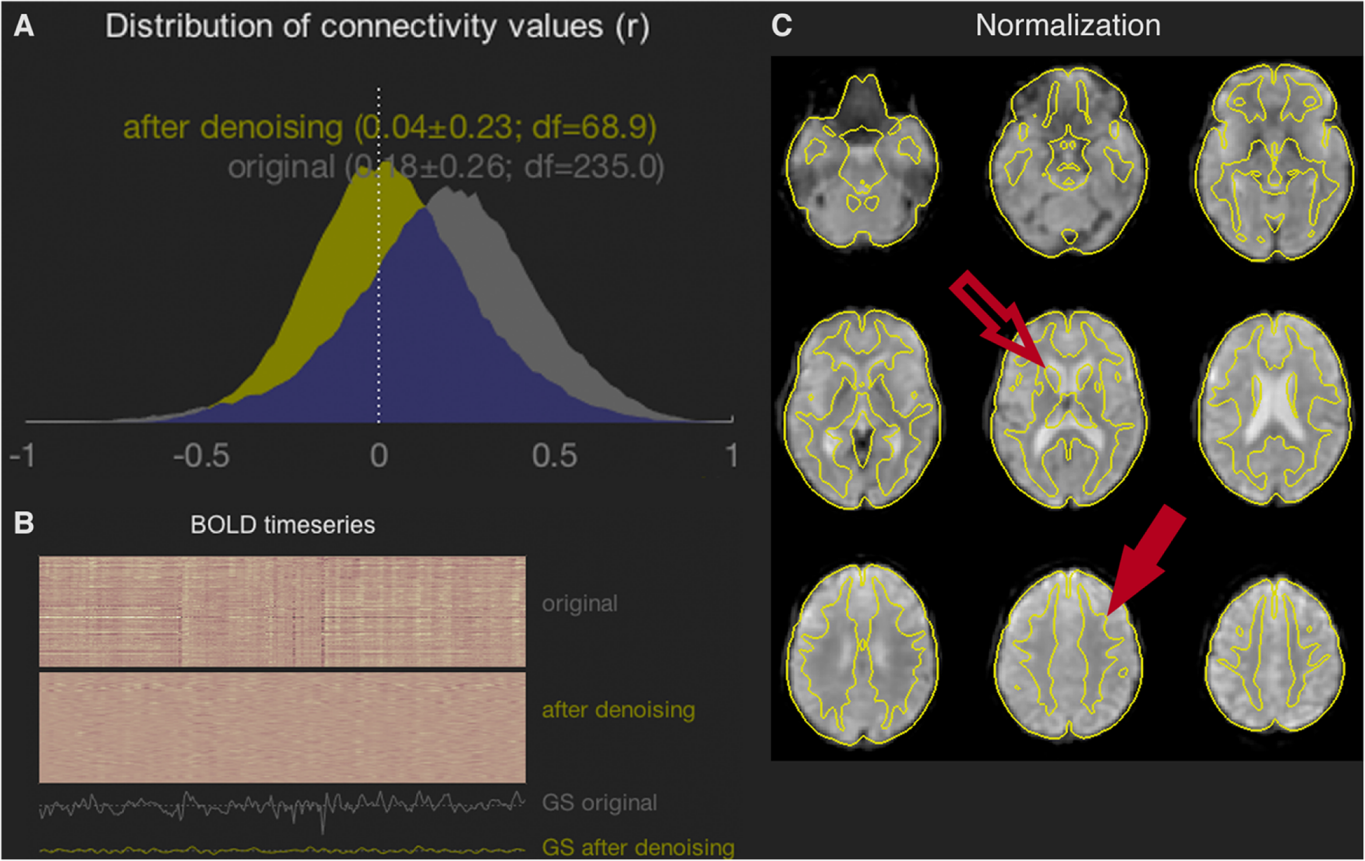
Data Quality Assessment After Denoising. All data comes from a single participant (participant 4), while the similar plots for the other 8 participants are shown in the supplemental material. No scrubbing was performed on this participant. **a** The distribution of connectivity values was shifted to have a mean value close to zero, and the SD was not overly broad compared to other participants in the study. **b** The carpet plot indicates several large spikes in the original data, likely representing motion artifacts, have been attenuated by the filtering operations applied. **c** The image shows BOLD data overlaid by grey-matter edges from the MNI standard brain, with excellent fidelity. For example: (i) the edges of the caudate nucleus (open arrow) are well situated compared to the lateral ventricles visible in the underlying image; (ii) image contrast is visible across the overlaid boundary in the superior frontal cortical region (closed arrow), indicating that the boundary follows the grey-matter/white-matter interface

We were able to scan and obtain good quality data from this group of young people with CP who demonstrated a range of GMFCS levels (Table 1). The distribution of connectivity values (r) pre and post-denoising retained acceptable shapes meeting our first criterion for good quality data. We are including one participant’s connectivity distributions (participant 4) as well as a carpet plot of the same participant’s BOLD time series as an example (Fig. 1a & b). For all other participants’ connectivity distributions and carpet plots see the supplementary material (supplementary Figure).

**Fig. 2:**
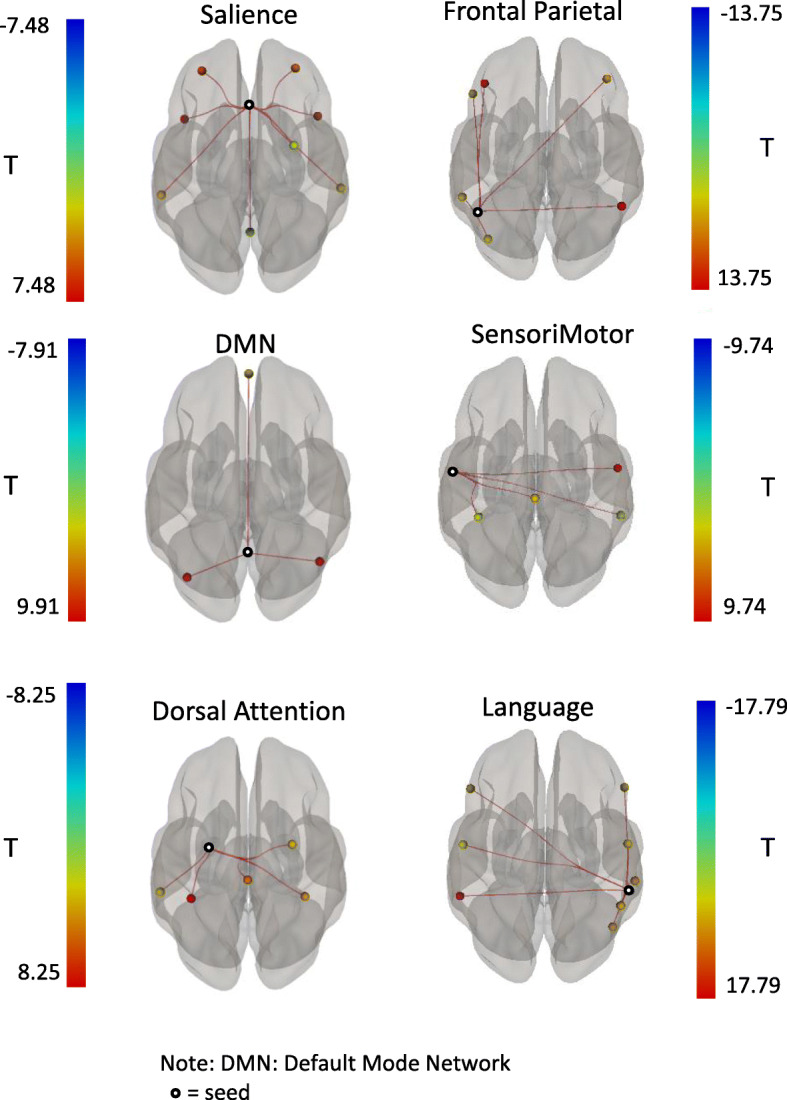
Within-Network Functional Connectivity. The regions of interest (ROIs) from the six networks examined in the present study are illustrated. Seed ROIs are indicated by a ring, while the colour of the other ROIs in each network represents the t-statistic associated with its correlation to the seed. The statistical values of the within-network connectivity are shown in Table 2

We were able to retain the full data set after motion scrubbing in majority of the participants (7 participants) while two participants (participant 7 &8) had 31 and 32 scrubbed volumes, leaving 205 and 204 time points, respectively. The participants who had volumes scrubbed retained around 6 min and 45 s of resting state data available for analyses meeting the second criterion for good quality data. These two participants did not present with more severe wellbeing or depressive symptoms but did report greater fatigue. One of the two participants also reported greater pain and impact of pain (supplementary Table). The functional connectivity networks of interest were also successfully identified in the data using standard seed regions and the significant correlations between the ROIs associated with the network (see Additional file 3) and the seed region (Fig. 2, Table 2).

**Table 2:** Statistical data associated with functional connectivity of the networks of interest

Seed	ROI (MNI coordinates)	beta	T(8)	p-FDR
**Salience Network**				
ACC (0,22,35)	PFC (L) (− 32,45,27)	.047	7.48	<.01
	PFC (R) (32,46,27)	0.42	5.48	<.01
	AInsula (R) (47,14,0)	0.52	4.83	.01
	AInsula (L) (−44,13,1)	0.61	5.55	<.01
	SMG (R) (62,-35,32)	0.26	4.22	.02
	SMG (L) (−60,-39,31)	.030	3.87	.02
**Default Mode Network**				
PCC (1,-61,38)	LP (R) (47,-67,29)	0.55	7.91	<.01
	LP (L) (−39,-77,33)	0.54	7.85	<.01
	MPFC (1,55,-3)	0.22	4.31	.02
**Dorsal Attention Network**				
FEF (L) (−27,-9,64)	IPS (L) (−39,-43,52)	0.62	8.25	<.01
	IPS (R) (39,-42,54)	0.34	4.15	.03
	FEF (R) (30,-6,64)	0.27	4.13	.03
**Frontal Parietal Network**				
PPC (L) (−46, −58, 49)	LPFC (R) (41, 38, 30)	0.31	4.63	.01
	LPFC (L) (−43, 33, 28)	0.59	10.83	<.01
	PPC (R) (52, −52, 45)	0.59	13.75	<.01
**SensoriMotor Network**				
Lateral (L) (−55, −12, 29)	Lateral (R) (56, −10, 29)	1.11	9.74	<.01
	Superior (0, −31, 67)	0.47	3.99	.03
**Language Network**				
pSTG (R) (59, −42, 13)	IFG (R) (54, 28, 1)	0.33	3.79	.02
	pSTG (L) (−57, −47, 15)	0.76	17.79	<.01
	IFG (L) (−51, 26, 2)	0.34	3.72	.02

The MNI coordinates are x,y,z coordinates in mm in standard MNI space

**Fig. 3:**
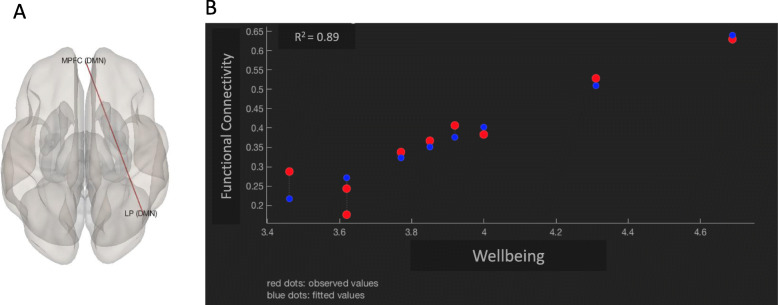
Correlation between functional connectivity and well-being. The image (**a**) identifies the locations of two functionally connected regions in the Default Mode Network (DMN): Medial Prefrontal Cortex (MPFC) and right Lateral Parietal (LP). The connectivity between these two regions was found to be highly correlated with well-being. The graph (**b**) displays the functional connectivity between MPFC and right LP and a linear regression with well-being. The high R-squared value of the regression indicates that 89% of the variance in functional connectivity is accounted for by the well-being measure

Our analyses also revealed FC patterns that were significantly associated with participants scores on Well-Being t(7) = 7.5, *p* = 0.004 (Fig. 2). This effect was further explored at the pairwise comparison level and showed significant associations between higher scores in well-being and higher levels of connectivity between the Medial PreFrontal Cortex (MPFC) and the right Lateral Parietal (LP) regions [*p* < 0.0001, *R*^2^ = 0.89] (Fig. 3). The FC patterns associated with depressive symptoms, pain severity, or impact of pain did not remain significant after FDR corrections for multiple comparisons.

## Discussion

The present study is the first to demonstrate that it is feasible to scan and obtain good quality resting state functional connectivity data in young adults with CP (18–29 years of age) with varying levels of gross motor ability (Table 1) without the use of sedation, given their motor and physical challenges and the associated expectations of fMRI scanning protocol. We were also able to identify the 6 networks of interest (Fig. 2, Table 2). These networks (DA, DMN, SN, the Frontal-Parietal network, the SensoriMotor network, and the Language network) have been previously identified in other groups. Our results show that the networks are also robustly represented in individuals with CP, given that all regions showed significant functional connectivity in the associated network. The fact that the within-network FDR-corrected *p*-values remained low also attests to the high quality of the acquired image data. Our analyses also revealed FC patterns that were significantly associated with levels of quality of life. The clinical significance and applicability of these observations are discussed below.

### Functional connectivity and well-being

Our results revealed a circuitry that was significantly correlated with reports of well-being. As FC between two functional nodes (MPFC and right LP) within the DMN increased, the reports of well-being also increased. Schacter et al. [52] have proposed the constructive episodic simulation hypothesis related to the “self” which holds that individuals formulate future representations by retrieving certain prior episodic memories and piecing them together to formulate a representation of what may take place in their personal future. The neural mechanism underlying this “episodic future thinking” has been linked to the regions that make up the default mode network [53] with particular regions having distinct roles. The MPFC has been associated with self-related processing including future goals, mental states, mental simulation of future self-related events [54, 55] and the lateral parietal cortex has been associated with “episodic memory retrieval”, self-related processing (representing the psychological aspect of the self) [54, 55], and “retrieval success” in memory activation [56]. There has also been some evidence suggesting that functional connectivity within the DMN (including medial frontal area) is associated with emotional intelligence, defined as “an individual’s capacity to accurately perceive, understand, reason about, and regulate emotions, and to apply that information to facilitate thought and achieve goals” [57].

A positive correlation between connectivity involving the MPFC and the LP associated with higher reports of well-being may index emotional understanding and greater capacity for adaptive emotion regulation. Emotion regulation (ER) is a set of processes employed to evaluate and modify emotional reactions, their intensity, and temporal duration to facilitate goal-directed behavior [58]. Adaptive ER strategies such as acceptance, problem solving, and cognitive reappraisal are effective in modifying emotional reactions and associated with lower levels of mental health symptoms [59] and stronger cognitive functioning [60]. This finding suggests that better socioemotional skills or adaptive ER skills may contribute to better quality of life in young adults with CP. The clinical implication of this finding may be that therapeutic efforts directed to strengthening prosocial and emotion regulation skills during the transition time between adolescence and young adulthood may help improve the perception of quality of life in young adults with CP.

## Limitations

The current study had a small sample size, as our aim was primarily to determine the feasibility of scanning youths and young adults with CP. As a result, many smaller effects may have been missed due to reduced power. In one analysis, we examined the association between connectivity patterns and clinical measures using single seeds and found several FDR corrected significant effects including associations between FC and depression. However, due to higher probabilities of type I error when running separate analyses, we opted for the more conservative f-test analysis by entering all 28 seeds simultaneously into the model. It would be important to replicate the current study in a larger sample examining the networks highlighted in the present study. Given the small sample size in this study, larger studies would have to be conducted before these results can be generalized to the larger population.

## Conclusion

The findings of the present study demonstrate that it is feasible to scan and obtain meaningful resting state functional connectivity data without the use of sedation and conduct group-level analysis in young adults with CP who had a range of gross motor abilities. Our results also identified a neural circuitry that appears to be affected as a result of reported quality of life. These findings identify these regions/circuitries as important seeds for further investigations into mental health and wellbeing.

## Supplementary information

**Additional file 1.** (A) Displays the distribution of connectivity values pre and post-denoising. (B) Shows the carpet plot for the BOLD series pre and post-denoising. (C) Reveals BOLD data overlaid by grey-matter edges from the MNI standard brain.

**Additional file 2.** Participants Clinical Scores. Note: Higher clinical scores indicate greater symptom severity except for wellbeing where higher scores reflect higher self reports of quality of life.

**Additional file 3.** The following table lists the regions of interest (ROIs) examined in this study, along with their (x,y,z) co-ordinates in MNI standard space.

## Data Availability

The data that support the findings of this study are available from the corresponding author upon reasonable request.

## Abbreviations

ACC: Anterior cingulate: salience
BOLD: Blood oxygen level dependent
BWpp: BandWidth per pixel (BWpp
CES-D: Center for Epidemiological Studies Depression Scale
CP: Cerebral palsy
CP-NET: Cerebral Palsy Integrated Neuroscience Discovery Network
CSF: CerebroSpinal Fluid
DA: Dorsal attention
DSM: Diagnostic and statistical manual
ER: Emotion regulation
FA: Flip angle
FC: Functional connectivity
FDR: False discovery rate
FEF: Frontal eye field
FISSA: Fatigue impact and severity self-assessment
fMRI: Functional magnetic resonance imaging
GM: Gray matter
GMFCS: Gross motor function classification system
IFG: Inferior frontal gyrus
IPS: Intra Partielta Sulcus
LP: Lateral parietal
LPFC: Lateral prefrontal cortex
LPFC: Prefrontal cortex
MPF: Medial prefrontal cortex
PCC: Posterior cingulate cortex
PPC: Posterior parietal cortex
pSTG: Posterior superior temporal gyrus
ROI: Region of Interest
RPFC: Rostral prefrontal cortex
SMG: Supra Marginal Gyrus
QoL: Quality of life
WM: White matter
