# Supplementary material for: Functional connectivity and quality of life in young adults with cerebral palsy: a feasibility study
Source: BMC Neurol. 2020 Oct 23;20:388. doi: 10.1186/s12883-020-01950-7 (PMC7583292; doi:10.1186/s12883-020-01950-7)
Supplement: Supplementary file 1 — Additional file 1. (A) Displays the distribution of connectivity values pre and post-denoising. (B) Shows the carpet plot for the BOLD series pre and post-denoising. (C) Reveals BOLD data overlaid by grey-matter edges from the MNI standard brain. [file 12883_2020_1950_MOESM1_ESM.pptx]

## Slide 1
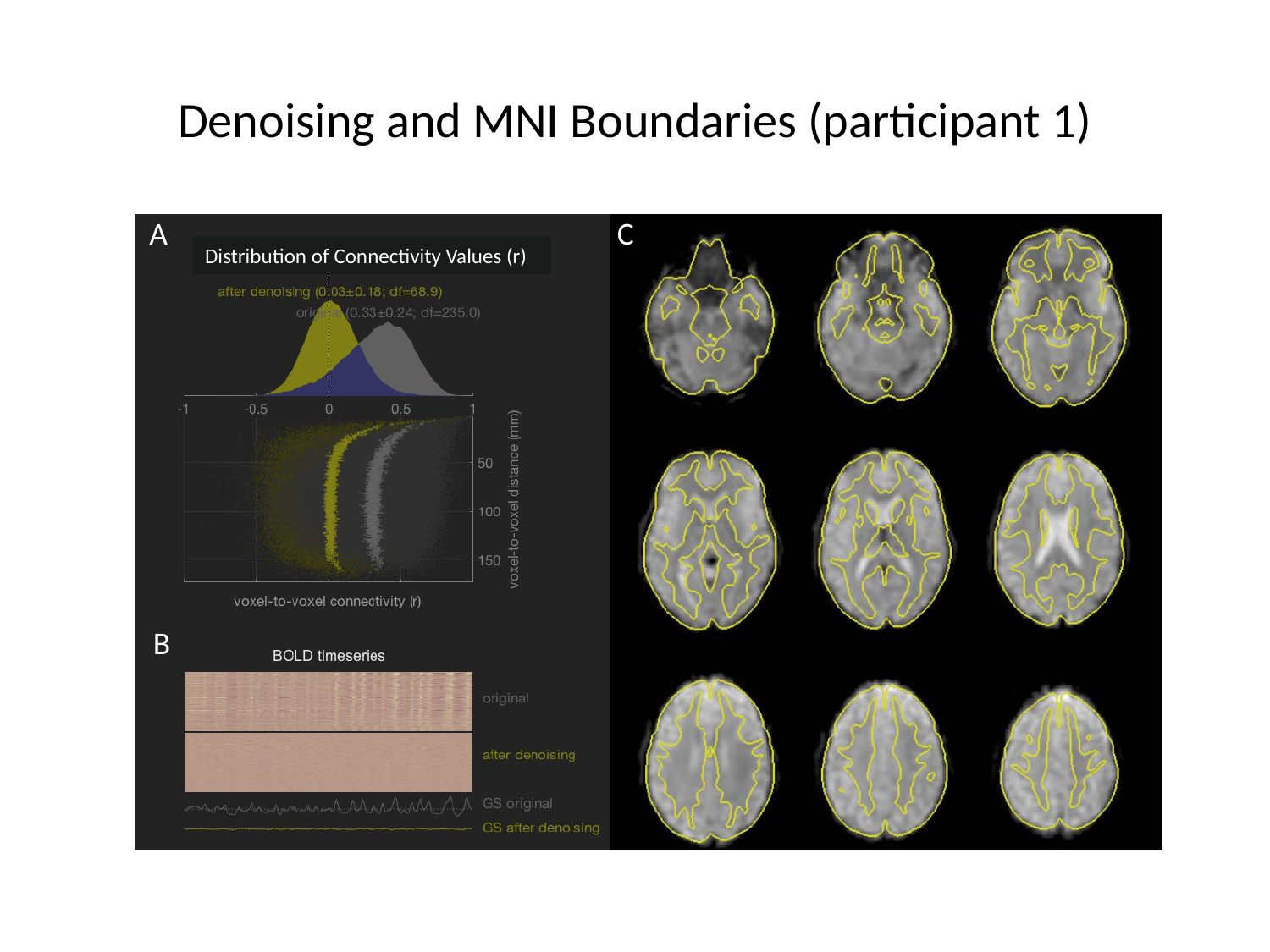

# Denoising and MNI Boundaries (participant 1)
A
C
Distribution of Connectivity Values (r)
B

## Slide 2
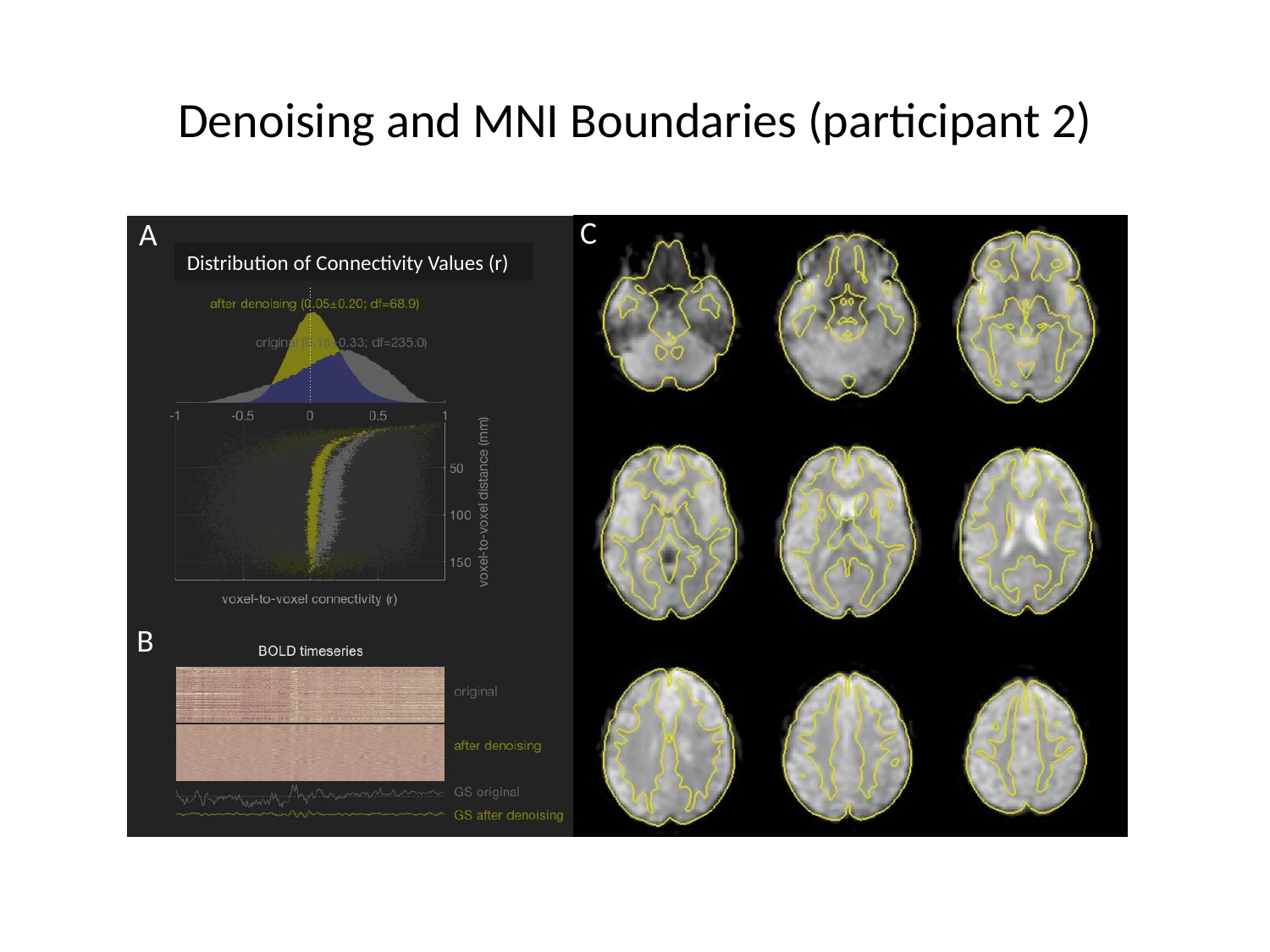

# Denoising and MNI Boundaries (participant 2)
C
A
Distribution of Connectivity Values (r)
B

## Slide 3
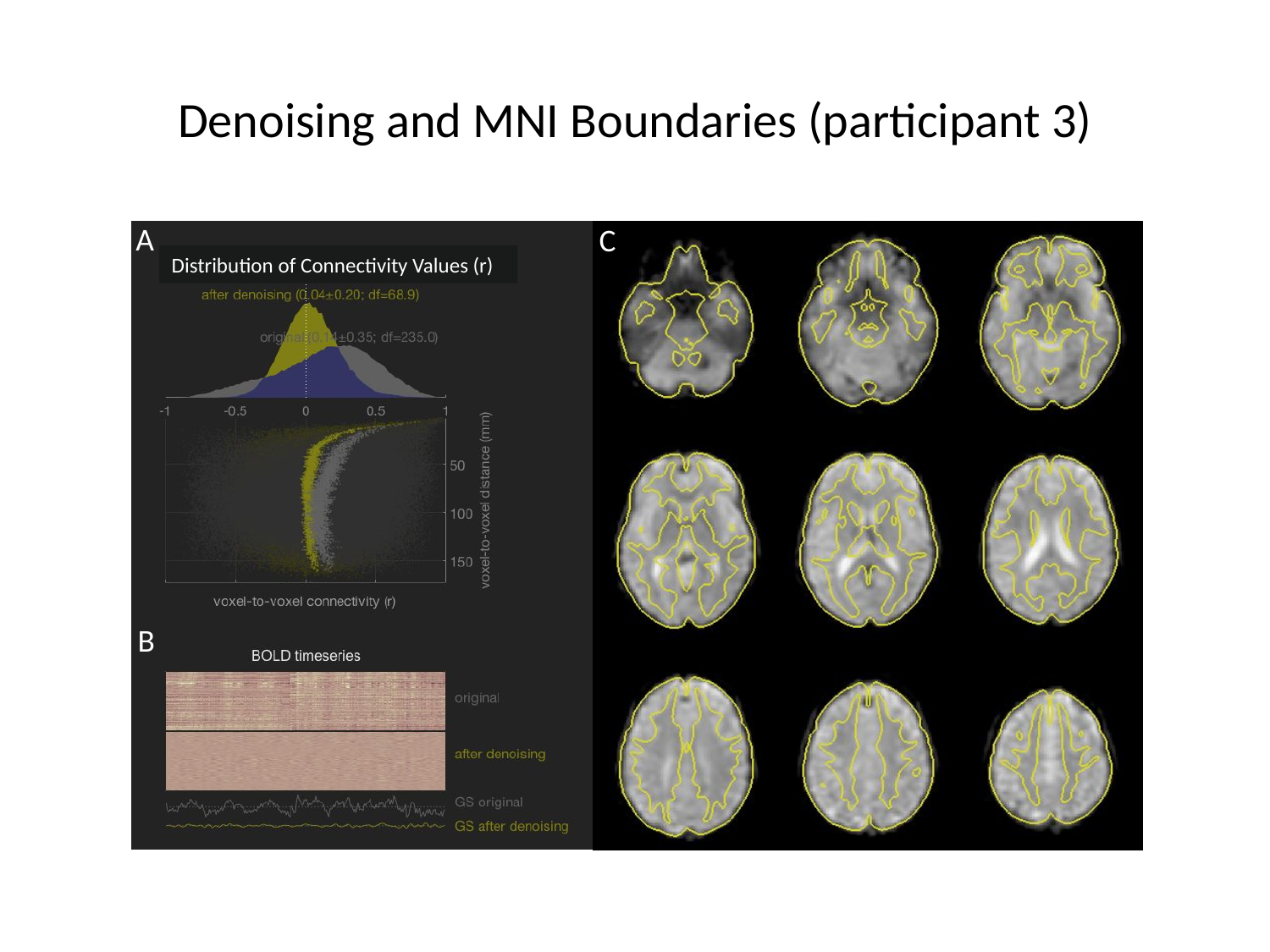

# Denoising and MNI Boundaries (participant 3)
A
C
Distribution of Connectivity Values (r)
B

## Slide 4
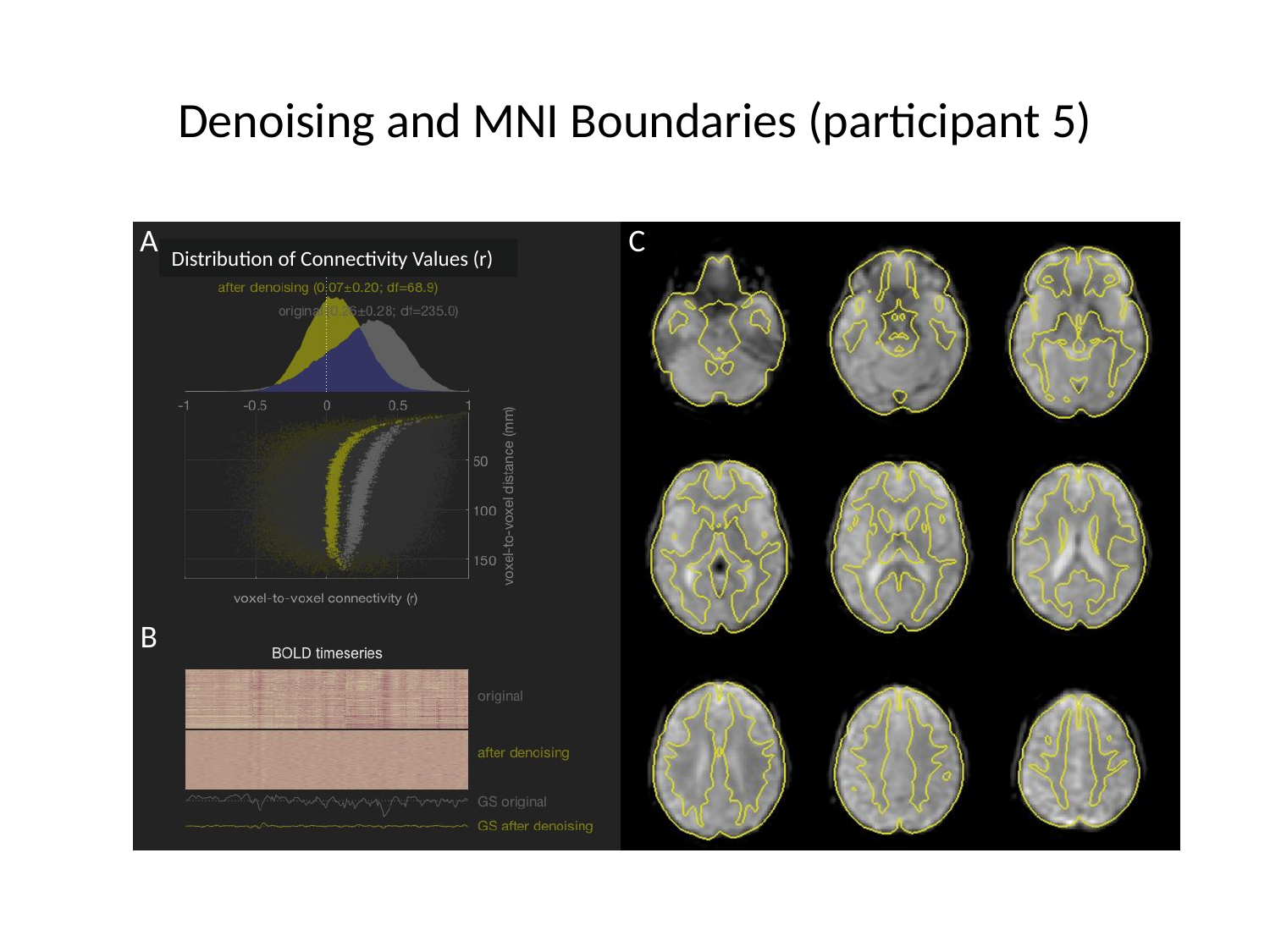

# Denoising and MNI Boundaries (participant 5)
C
A
Distribution of Connectivity Values (r)
B

## Slide 5
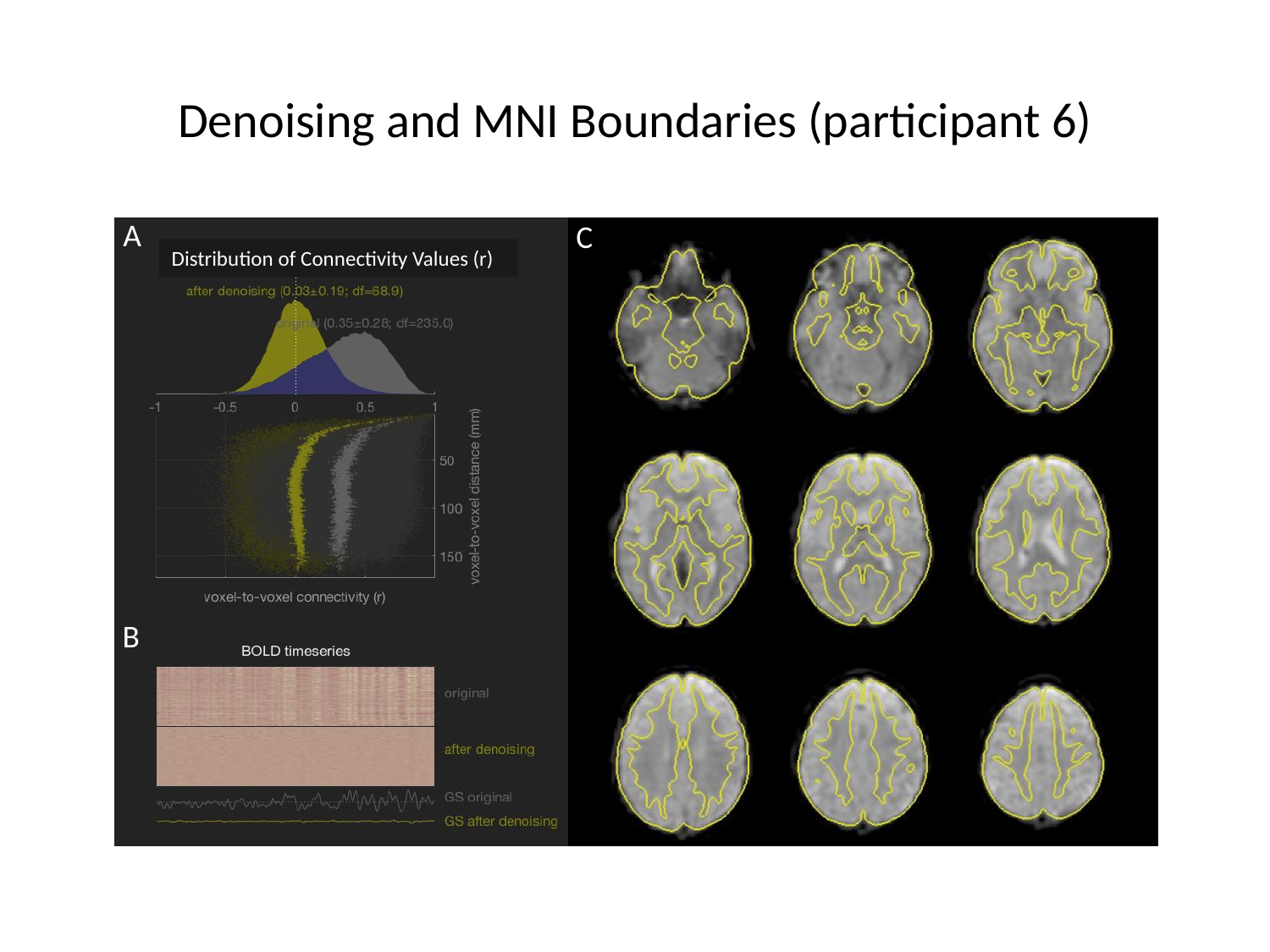

# Denoising and MNI Boundaries (participant 6)
A
C
Distribution of Connectivity Values (r)
B

## Slide 6
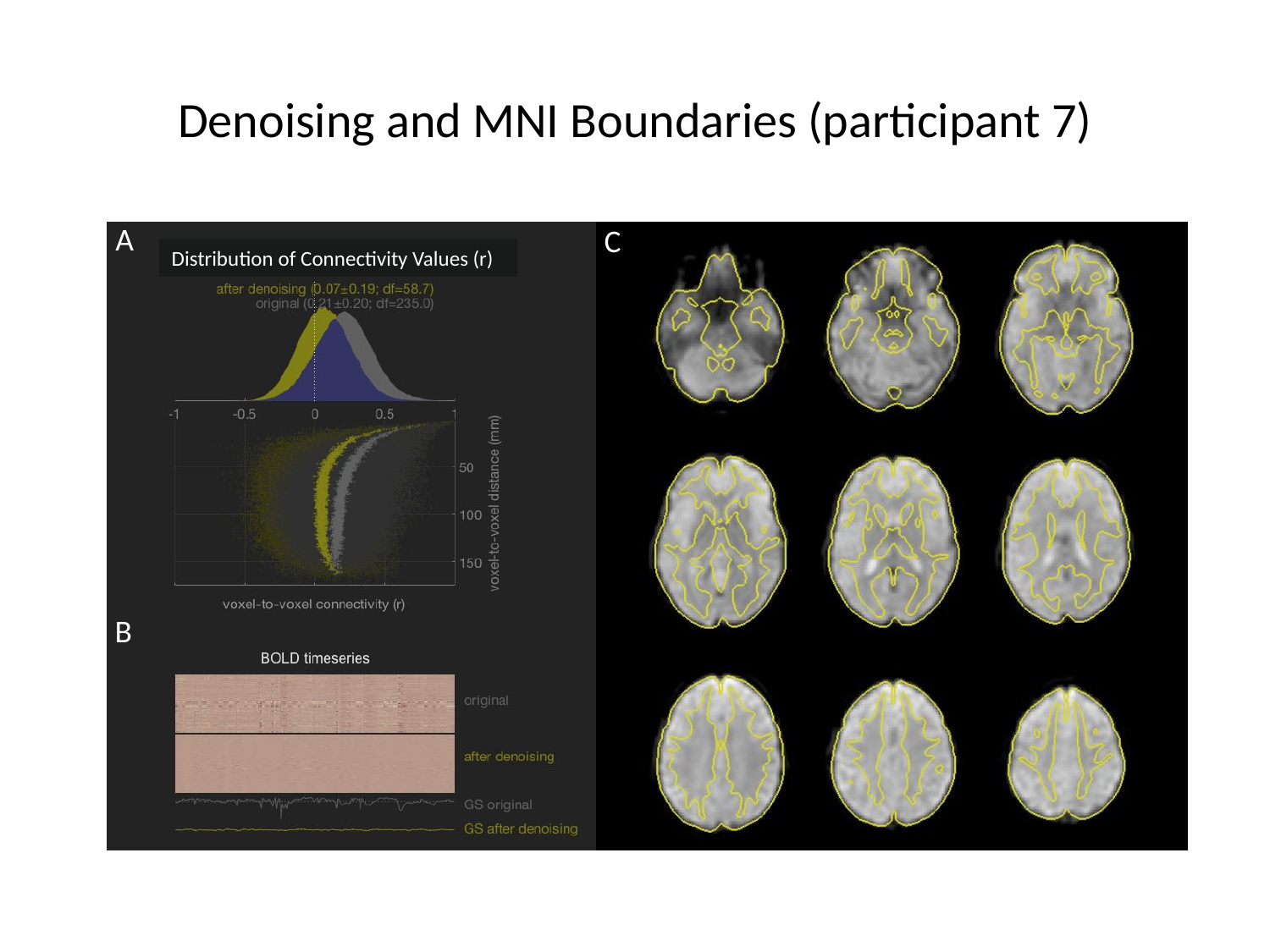

# Denoising and MNI Boundaries (participant 7)
A
C
Distribution of Connectivity Values (r)
B

## Slide 7
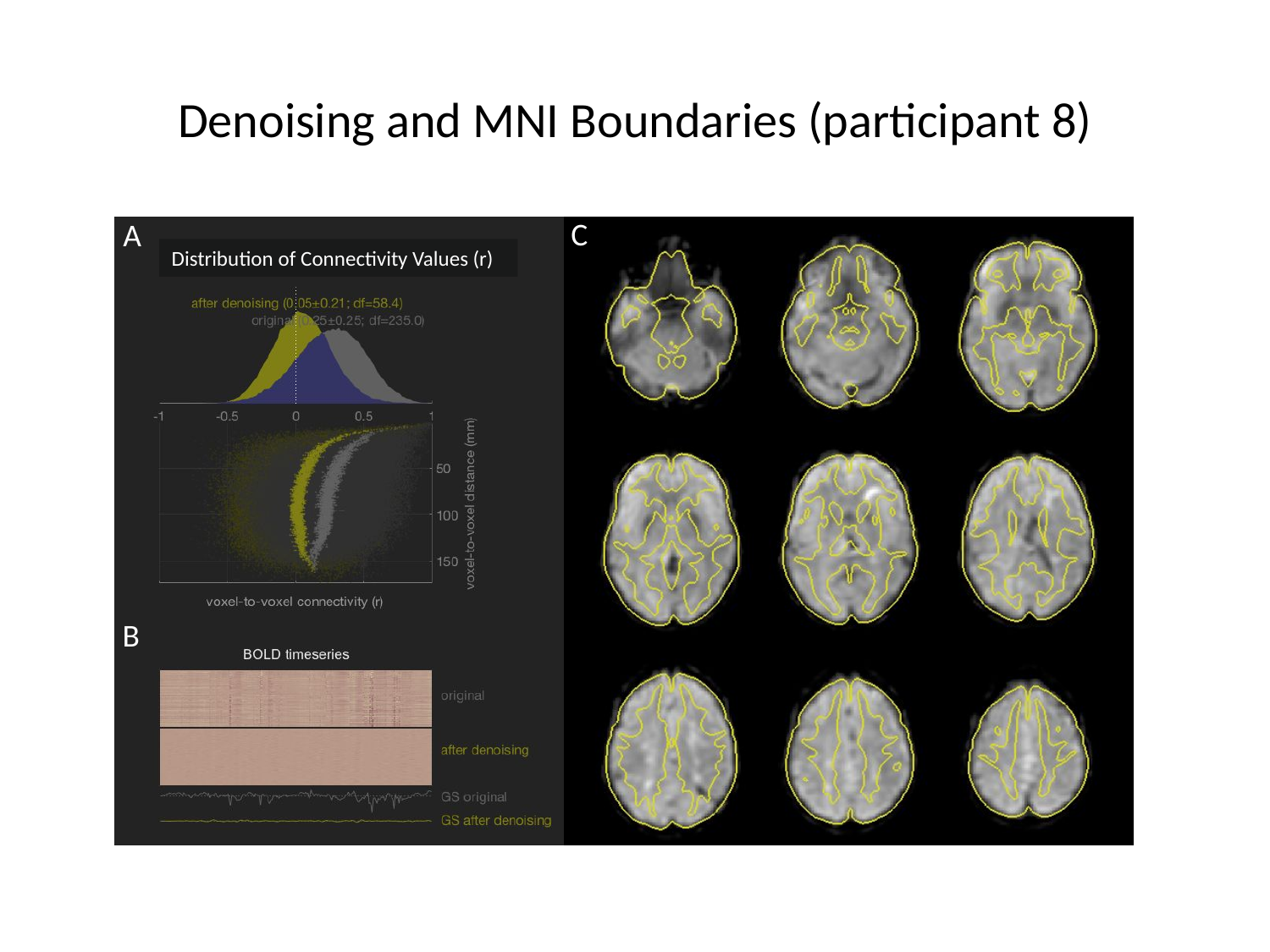

# Denoising and MNI Boundaries (participant 8)
C
A
Distribution of Connectivity Values (r)
B

## Slide 8
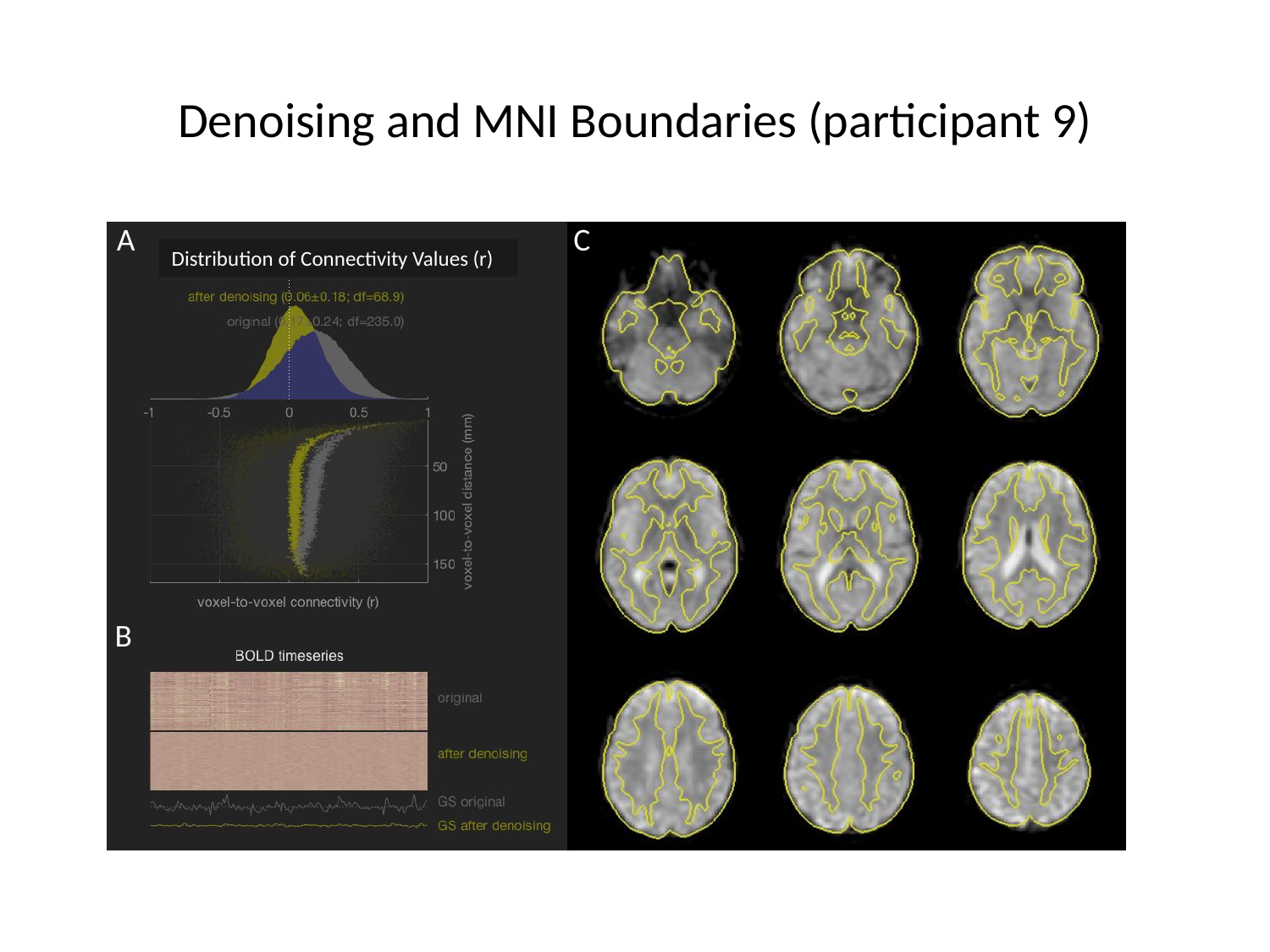

# Denoising and MNI Boundaries (participant 9)
A
C
Distribution of Connectivity Values (r)
B
