# Supplementary material for: Functional connectivity and quality of life in young adults with cerebral palsy: a feasibility study
Source: BMC Neurol. 2020 Oct 23;20:388. doi: 10.1186/s12883-020-01950-7 (PMC7583292; doi:10.1186/s12883-020-01950-7)
Supplement: Supplementary file 2 — Additional file 2. Participants Clinical Scores. Note: Higher clinical scores indicate greater symptom severity except for wellbeing where higher scores reflect higher self reports of quality of life. [file 12883_2020_1950_MOESM2_ESM.docx]

Supplementary Table.

*Participants Clinical Scores*

| **Participant** | **WellBeing** | **Depressive Severity** | **Fatigue** | **Pain Impact** | **Pain Severity** |
| --- | --- | --- | --- | --- | --- |
| 1 | 3.46 | 37 | 56 | 1 | 2 |
| 2 | 3.62 | 40 | 113 | 7 | 7 |
| 3 | 4.69 | 39 | 73 | 1 | 1 |
| 4 | 3.77 | 52 | 87 | 5 | 5 |
| 5 | 3.85 | 38 | 58 | 1 | 1 |
| 6 | 4.00 | 29 | 78 | 4 | 4 |
| 7 | 4.31 | 26 | 111 | 5 | 6 |
| 8 | 3.62 | 24 | 122 | 10 | 9 |
| 9 | 3.92 | 53 | 57 | 4 | 7 |

Note: Higher clinical scores indicate greater symptom severity except for wellbeing where higher scores reflect higher self reports of quality of life.
