# Supplementary material for: Functional connectivity and quality of life in young adults with cerebral palsy: a feasibility study
Source: BMC Neurol. 2020 Oct 23;20:388. doi: 10.1186/s12883-020-01950-7 (PMC7583292; doi:10.1186/s12883-020-01950-7)
Supplement: Supplementary file 3 — Additional file 3. The following table lists the regions of interest (ROIs) examined in this study, along with their (x,y,z) co-ordinates in MNI standard space. [file 12883_2020_1950_MOESM3_ESM.docx]

**Additional file 3**

The following table lists the regions of interest (ROIs) examined in this study, along with their (x,y,z) co-ordinates in MNI standard space.

| **ROI** | **MNI Co-ords**  **(x, y, z) (mm)** | **Full Name** |
| --- | --- | --- |
| **Default Mode Network** | | |
| MPFC | (1, 55, -3) | Medial Prefrontal Cortex |
| LP (L) | (-39, -77, 33) | Left Lateral Parietal |
| LP (R) | (47, -67, 29) | Right Lateral Parietal |
| PCC | (1, -61, 38) | Posterior Cingulate Cortex |
| **SensoriMotor Network** | | |
| Lateral (L) | (-55, -12, 29) | Left Lateral Central |
| Lateral (R) | (56, -10, 29) | Right Lateral Central |
| Superior | (0, -31, 67) | Superior Central |
| **Salience Network** | | |
| ACC | (0, 22, 35) | Anterior Cingulate Cortex |
| AInsula (L) | (-44, 13, 1) | Left Anterior Insula (Frontal Operculum) Cortex |
| AInsula (R) | (47, 14, 0) | Right Anterior Insula (Frontal Operculum) Cortex |
| RPFC (L) | (-32, 45, 27) | Left Rostral Prefrontal Cortex |
| RPFC (R) | (32, 46, 27) | Right Rostral Prefrontal Cortex |
| SMG (L) | (-60, -39, 31) | Left Supra Marginal Gyrus |
| SMG (R) | (62, -35, 32) | Right Supra Marginal Gyrus |
| **Dorsal Attention Network** | | |
| FEF (L) | (-27, -9, 64) | Left Frontal Eye Field |
| FEF (R) | (30, -6, 64) | Right Frontal Eye Field |
| IPS (L) | (-39, -43, 52) | Left Intra Parietal Sulcus |
| IPS (R) | (39, -42, 54) | Right Intra Parietal Sulcus |
| **Frontal Parietal Network** | | |
| LPFC (L) | (-43, 33, 28) | Left Lateral Prefrontal Cortex |
| LPFC (R) | (41, 38, 30) | Right Lateral Prefrontal Cortex |
| PPC (L) | (-46, -58, 49) | Left Posterior Parietal Cortex |
| PPC (R) | (52, -52, 45) | Right Posterior Parietal Cortex |
| **Language Network** | | |
| IFG (L) | (-51, 26, 2) | Left Inferior Frontal Gyrus |
| IFG (R) | (54, 28, 1) | Right Inferior Frontal Gyrus |
| pSTG (L) | (-57, -47, 15) | Left Posterior Superior Temporal Gyrus |
| pSTG (R) | (59, -42, 13) | Right Posterior Superior Temporal Gyrus |
| **Cerebellar** | | |
| Cerebellar.Anterior | (0, -63, -30) | Anterior Cerebellum |
| Cerebellar.Posterior | (0, -79, -32) | Posterior Cerebellum |
